# A preliminary evaluation of psychological stress amongst workers in Taiwan: a cross-sectional survey

**DOI:** 10.1186/s13033-019-0290-y

**Published:** 2019-05-16

**Authors:** Wan-Yu Yeh, Wei-Che Chiu, Ruey-Yu Chen, Pei-Yi Hu, Dung-Min Tsai

**Affiliations:** 1 Center for Evidence-Based Medicine, Taipei Veterans General Hospital, 201, Sec. 2, Shipai Rd., Beitou District, Taipei, Taiwan, ROC; 2 Department of Health Administration, Tzu Chi University of Science and Technology, 880 Sec.2, Chien-Kuo Rd., Hualien, Taiwan, ROC; 3 Department of Psychiatry, Cathay General Hospital, 280 Renai Rd., Sec.4, Taipei, Taiwan, ROC; 4 School of Medicine, College of Medicine, Fu-Jen Catholic University, 510 Zhongzheng Rd., Xinzhuang Dist., New Taipei, Taiwan, ROC; 5 School of Public Health, Taipei Medical University, 250 Wuxing St., Taipei, Taiwan, ROC; 6 Division of Labor Relations, Institute of Labor Occupational Safety and Health, Ministry of Labor, 99, Lane 407, Hengke Rd., Sijhih District, New Taipei, Taiwan, ROC

**Keywords:** Work stress, Mental illness, Occupational disease, Reference guide on identification, Stressful life events, Taiwan

## Abstract

**Background:**

Stress and psychological disorders have been assigned increasing significance in the field of occupational health. Based on Japan’s psychiatric disability occupational disease recognition regulation, Taiwan’s Council of Labor Affairs announced “Evaluation Guidelines for psychiatric diseases induced by work-related stress” in 2009. This evaluation tool was designed to assess the source and intensity of work-related and non-work-related mental stress, and references existing Japanese guidelines. However, empirical data from workers in various sectors in Taiwan are still required to validate the utility of the guidelines.

**Methods:**

This study recruited 2319 workers from the manufacturing, service, and public administration sectors to participate in a survey between 2010  and  2011. The survey included questions regarding participants’ demographic characteristics, job type or attributes, a life event stress intensity evaluation Table (35 work-related and 23 non-work-related items on a scale of 1–10). The Chinese version of the Copenhagen Burnout Inventory (C-CBI) and Chinese Health Questionnaire (CHQ-12) were also included to explore associations between work-related/non-work-related stress and health outcomes.

**Results:**

Analyses of survey results showed events relating to employment security (e.g., “company bankruptcy” and “being fired or forced to retire” scores; mean stress intensity scores both 6.18) were the cause of the highest intensity work-related stress. Within different demographic/job type categories, women had higher stress intensity scores for most items than men (greatest difference in “sexual harassment in the workplace” score). Furthermore, executive class workers generally experienced more psychological stress than blue-collar workers (greatest difference in “serious injury or disease due to work” score). Results from regression analysis supported the observation that employees’ burnout and work-related stress was more significant than non-work-related stress. Moreover, work-related/non-work-related stress intensity levels both had significant negative predictive effects on mental health.

**Conclusions:**

Regarding policy, this study provides empirical evidence and practical suggestions for establishing a psychological stress intensity database of workers under specific social contexts in a newly industrialized East Asian country. Such a database can be employed to help identify workers with work-related psychological disorders. Additionally, this study also provides a point of reference for enterprises to prioritize agendas when developing employee stress management and support protocols.

## Background

Occupational health has traditionally focused on hazardous work environments in industrial settings. However, with rapid changes in industrial economics and employment patterns, occupational health now also encompasses work distribution within organizations and employees’ psychosocial status. In addition, it focusses on relevant work-related stressors, such as work amount, pace, time, organizational ability, interpersonal relationships, control over work content, and unstable employment. Combined, these elements can impact workers’ mental health and are considered emerging occupational health threats.

Until now, international opinion on occupational disease compensation for workers experiencing work-related psychological disorders remains unclear and highly controversial. In countries such as the United States, the United Kingdom, Canada, and Australia, numerous court cases have recognized litigation precedents, attempting to clarify the scope of a company’s social responsibility in protecting employee’s psychological health [1–5]. Japan has been an international leader in benchmarking and the statistical analysis of work-related health measures. In addition to “Karoshi (a Japanese term meaning ‘death from overwork’)”, Japan has also recently experienced numerous cases of work-related mental illnesses and psychiatric disabilities (such as depression, psychosomatic disorder, and suicide). In 1999, based on the “Labor Standards Act Enforcement Rules,” article 35, paragraph 9, regarding “definitive work-related diseases that result from other causes,” “Guidelines on the Applicability of Workers’ Compensation to Patients with Mental Disorders Related to Psychological Load” (hereafter referred to as Japanese evaluation guidelines) were announced [6]. These evaluation guidelines included “psychological disorders caused by work-related stress.” Integrated and coordinated measures were established for the elements of regulations, acknowledgment or recognition, and executive administration. Diagnostic or recognition technologies, as well as practical application principles, were also meticulously developed. In Japan, the definitive diagnosis or recognition of “psychological disorders caused by professional work” involves 3 verification procedures after a confirmed psychiatric episode. First, the stress intensities of “professional work-related stress events” were defined. Then, “non-work-related” stress levels were noted. The background and medical history of the individual were then considered. In the absence of high non-work-related stress, a psychiatric disorder history, addictive drug usage, and other personal factors leading to stress, if an individual experienced work-related stress events at a 3-level intensity (where Level I is weak intensity, Level II is moderate, and Level III is severe) 6 months before a psychiatric episode, the psychological disorder can be considered work-related. To verify individual cases, data such as a list of stressful life events and the population mean stress intensity score for each event is documented in a mental stress evaluation table for work-related and non-work-related environments [7–10].

Following Japan’s evaluation guidelines, evaluations of the source and intensity of an individual’s work-related and non-work-related stress should be based primarily on stressful life events (SLEs), which include a list of possible negative/positive stressful situations (including major life events as well as daily hassles) that people may experience in everyday life in or outside of the workplace [8, 9]. Analysis of the research data led to the determination of an objective group average of the stress intensity for these life events. This method was based on the “Social Readjustment Rating Scale (SRRS)” proposed by American psychiatrists Holmes and Rahe [11]. When developing this scale, norms were established by investigating 5000 patients. The 43 most frequently occurring stress events of everyday life, including “spousal death” and “serious negligence at work,” were chosen. The total score for major life events a person experienced in the preceding year represented the stress standard they experience. The concept of the SLE method is relatively simple. This method recognizes that environmental events bring about a certain stress standard, and the effect of these events is accumulative. When the accumulated stress standard of the events exceeds an individual’s ability to withstand or adjust, the individual may experience feelings of burnout or even develop stress-related symptoms or diseases. To increase the scope of the scale for work-related life events, academics recruited by Japan’s evaluation guideline amendment team added a further 18 common stress sources for workers to the original SRRS for a labor stress investigation table that contains 65 items [8]. They also instructed interviewees to use their experience and imagination when rating the stress intensity of each work-related or non-work-related event. These results could then be employed to establish a mental stress intensity database for Japanese workers. Following consultations and conferences with experts and its application in numerous large-scale workplace surveys, this mental stress evaluation table for stressors in and outside of the workplace was considered appropriate for use in Japan. However, the table still undergoes continual monitoring and adjustment to support the changing trends of the Japanese workplace. Since the evaluation guidelines were initially announced in 1999, the stress event items listed in the evaluation guidelines have undergone various amendments. The most recent amendment was announced in December 2011 [12].

Of the numerous theoretical models for work-related stress, the SLE method is unique in that it is conceptually clear and easy to operate. The SLE method has received good ratings by international research societies for work-related stress. The SRRS can be relatively easily adjusted to various ethnicities/regions based on the characteristics of the research targets [9, 10, 13]. Additionally, a number of studies have indicated that a correlation exists between SLE score (measured by SRRS or revised measurement) and depression, anxiety, neuroticism or even suicidal behavior [14–18]. Therefore, the SRRS is a valuable individual stress evaluation tool for recognizing work-related psychological disorders.

After researching the above-mentioned techniques and the experiences of Japan, Taiwan’s Council of Labor Affairs announced the “Evaluation Guidelines for psychiatric diseases induced by work-related mental stress” (hereafter referred to as Taiwanese evaluation guidelines) in 2009 [19]. Then in 2010, psychological disorders were included in statistical items for occupational diseases. In the initial stage of policy development, Taiwan referenced work-related and non-work-related SLEs from those established in Japan (Tables 1, 2 list the recognized stress event items included in this study). The use of a Japanese evaluation tool to assess the mental stress experiences of workers in Taiwan may require revision because of the differences in industry patterns, lifestyles, and cultural beliefs between workers in the 2 countries. Therefore, in this study, we employed the Taiwanese evaluation guidelines and their work-related/non-work-related mental stress evaluation tool and modified them into a self-reported scale. The objective of this research study was to address an unmet need in the objective assessment of mental stress amongst workers in the Taiwanese population. It is suggested that this data may then provide a basis for the evaluation of work-related psychological disorders that is relevant to the compensation system in Taiwan’s social context.

**Table 1:** Variance analysis on gender, age and employment grade of 35 work-related stress intensity item mean scores (n = 2319)

Work-related stress events	Stress intensity mean	Stress intensity rank	Gender		*p*	${\text{r}}^{ 2}_{\text{pbi}}$	Age group			*p*	Epsilon^2^	Employment grade^a^			*p*	Epsilon^2^
			Mean (M)	Mean (F)			Mean (< 30)	Mean (30–40)	Mean (≧ 40)			Mean (Grade 1&2)	Mean (Grade 3&4)	Mean (Grade 5&6)		
Serious injury or disease due to work (including accidents during commuting)	5.39	12	4.87	6.14	***	0.039	5.33	5.22	5.79	***	0.005	5.93	5.43	5.03	***	0.024
Experienced (witnessed) miserable accidents or disasters in workplace	4.80	25	4.30	5.51	***	0.044	4.76	4.61	5.18	***	0.006	5.06	4.85	4.58	**	0.020
Occupational accident due to own acts (including serious personal injuries and traffic accidents etc.)	5.47	10	4.89	6.29	***	0.044	5.53	5.24	5.84	***	0.005	5.93	5.67	5.02	***	0.023
Serious negligence which influences the operation of company	5.53	8	4.96	6.32	***	0.046	5.54	5.36	5.85	**	0.003	6.02	5.70	5.07	***	0.012
Being investigated about your responsibility to accidents (events) in company	5.64	6	5.03	6.48	***	0.057	5.66	5.46	5.94	**	0.004	6.13	5.77	5.22	***	0.015
Being demanded to do illegal behaviors such as tax evasion	5.39	13	4.66	6.41	***	0.063	5.55	5.12	5.75	***	0.006	5.92	5.59	4.91	***	0.008
Significant financial losses at work	5.69	5	4.89	6.69	***	0.068	5.83	5.47	6.02	**	0.005	6.41	5.80	5.19	***	0.000
Being assigned a task seems to be unachievable	5.44	11	4.83	6.29	***	0.070	5.52	5.25	5.71	**	0.005	5.96	5.59	5.02	***	0.002
Could not achieve the assigned work goal	5.48	9	4.96	6.21	***	0.052	5.68	5.30	5.64	**	0.003	6.03	5.64	5.02	***	0.000
Became an executive for a new venture or company reorganization	5.17	17	4.62	5.94	***	0.050	5.21	5.03	5.41	*	0.002	5.59	5.30	4.81	***	0.001
Unreasonable demands or claims from customers or clients	5.09	19	4.46	5.96	***	0.068	5.25	4.80	5.44	***	0.009	5.66	4.98	4.81	***	-0.001
Being requested to deliver speeches or presentations in a large session or a formal occasion	4.99	21	4.42	5.78	***	0.059	5.09	4.83	5.23	*	0.003	5.36	5.05	4.72	***	0.001
Being appointed to be a deputy of superior	4.63	30	4.13	5.33	***	0.052	4.87	4.42	4.78	***	0.005	4.74	4.65	4.54	ns	
Major changes in contents or loading of work	4.85	23	4.44	5.40	***	0.038	5.05	4.69	4.95	**	0.003	5.06	4.79	4.79	ns	
Obvious increase of working hours	4.76	26	4.36	5.31	***	0.034	5.03	4.70	4.65	*	0.003	4.88	4.70	4.74	ns	
Work environment automation and computerization	3.46	35	3.18	3.81	***	0.018	3.32	3.31	3.82	***	0.009	3.56	3.30	3.53	ns	
Being required to shift or transfer work shift	4.30	34	3.90	4.84	***	0.032	4.21	4.33	4.34	ns		4.29	4.28	4.33	ns	
Company bankruptcy	6.18	1	5.88	6.59	***	0.012	5.86	6.10	6.65	***	0.008	6.32	6.24	6.01	ns	
Being fired or forced to retire	6.18	1	5.76	6.77	***	0.024	6.01	5.99	6.66	***	0.008	6.48	6.29	5.88	***	0.006
Transfer to other branch companies	5.06	20	4.59	5.73	***	0.041	4.97	4.87	5.46	***	0.007	5.28	5.12	4.86	*	0.003
Demotion	5.27	16	4.84	5.86	***	0.030	5.41	5.06	5.49	**	0.004	5.57	5.39	4.95	***	0.007
Promotion (thus change the work responsibility)	4.49	31	4.11	5.00	***	0.032	4.45	4.47	4.55	ns		4.62	4.53	4.35	ns	
Encounter discrimination or disbenefit because of non-regular employee status	4.75	27	4.28	5.41	***	0.041	4.90	4.58	4.91	*	0.003	4.96	4.83	4.57	*	0.003
Transfer to other places (Be transferred to nonlocal places)	4.82	24	4.28	5.56	***	0.051	4.70	4.68	5.16	***	0.005	5.09	4.89	4.58	***	0.005
Take the responsibility for a job, which was charged by several employees but by yourself now	5.37	15	4.94	5.96	***	0.034	5.75	5.16	5.37	***	0.007	5.40	5.36	5.36	ns	
Be transferred	4.68	28	4.27	5.24	***	0.035	4.68	4.53	4.94	**	0.004	4.92	4.62	4.57	*	0.002
Increase or decrease of subordinates	4.37	32	4.00	4.87	***	0.031	4.39	4.31	4.40	ns		4.59	4.24	4.35	*	0.002
Suffer from severe unreasonable or ill treatment, humiliation, or violent behavior	5.62	7	4.95	6.56	***	0.072	5.86	5.39	5.78	**	0.004	5.88	5.79	5.37	***	0.005
Sexual harassment	4.99	22	3.88	6.54	***	0.161	5.56	4.60	5.14	***	0.014	5.27	5.20	4.68	***	0.006
Dispute with superiors	5.37	14	4.67	6.35	***	0.079	5.60	5.18	5.51	**	0.003	5.71	5.57	5.06	***	0.009
Dispute with colleagues	5.10	18	4.49	5.94	***	0.068	5.52	4.90	5.06	***	0.008	5.34	5.20	4.90	**	0.004
Dispute with subordinates	4.64	29	4.10	5.40	***	0.054	4.94	4.51	4.64	*	0.003	4.88	4.74	4.44	**	0.004
Direct management change	4.35	32	3.90	4.97	***	0.042	4.50	4.21	4.47	*	0.002	4.63	4.43	4.12	***	0.006
Unpaid leave/leave of absence^b^	5.85	4	5.66	6.10	***	0.005										
Uncertain career prospects^b^	6.07	3	5.92	6.27	**	0.004										

*p < 0.05; **p < 0.01; ***p < 0.001; ns: not significant

${\text{r}}^{ 2}_{\text{pbi}}$: Square of point-biserial correlation coefficient between “gender” and stress intensity item mean scores; Epsilon^2^: Explanation variation of “age group” and “employment grade” and stress intensity item mean scores according to the calculation formula Epsilon^2^ = dfb(F − l)/dfb + dfw, derived from Hobson et al. [13]

^a^Grade 1: administrators and managers; Grade 2: professionals; Grade 3: non-manual skilled; Grade 4: non-manual low-skilled; Grade 5: manual skilled; Grade 6: manual low-skilled

^b^New suggested items through the expert meeting conducted by the researchers. Not included in Taiwan’s“Evaluation Guidelines for psychiatric diseases induced by work-related mental stress”

**Table 2:** Variance analysis on gender, age and employment grade of 23 non-work-related stress intensity item mean scores (n = 2319)

Non-work-related stress events	Stress intensity mean	Stress intensity rank	Gender		*p*	${\text{r}}^{ 2}_{\text{pbi}}$	Age group			*p*	Epsilon^2^	Employment grade^a^			*p*	Epsilon^2^
			Mean (Male)	Mean (Male)			Mean (< 30)	Mean (30–40)	Mean (≧40)			Mean (Grade 1 and 2)	Mean (Grade 3 and 4)	Mean (Grade 5 and 6)		
Get married	4.81	19	4.73	4.92	ns		5.20	4.96	4.24	***	0.017	4.83	4.83	4.82	ns	-0.001
Income decrease	6.62	4	6.37	6.96	***	0.012	6.83	6.67	6.35	**	0.004	6.55	6.58	6.75	ns	0.000
Own serious injury or abortion (need surgery, hospitalization or care tracks)	6.14	8	5.67	6.82	***	0.033	6.28	5.97	6.40	*	0.003	6.66	6.28	5.72	***	0.015
Divorce or separation	5.77	13	5.21	6.34	***	0.033	5.82	5.74	5.80	ns		6.28	5.79	5.34	***	0.014
Pregnancy (own or mate’s)	4.85	18	4.48	5.27	***	0.020	5.49	4.79	4.50	***	0.017	5.02	4.93	4.71	ns	0.001
Quarrel with spouse or serious problems of intimate relationships	5.83	12	5.34	6.32	***	0.027	5.96	5.88	5.70	ns		6.19	5.87	5.54	**	0.007
Serious injury, disease or health degeneration to close families	6.93	1	6.33	7.59	***	0.049	7.18	6.75	7.02	*	0.003	7.30	7.03	6.55	***	0.012
Death of a spouse, children, parents or sibling	6.87	2	6.04	7.71	***	0.070	7.29	6.70	6.80	**	0.005	7.45	6.88	6.43	***	0.017
Severe difficulties with immediate family members (including parental/marital relationship)	6.31	7	5.81	6.86	***	0.033	6.61	6.20	6.28	ns		6.70	6.36	5.99	***	0.010
Dishonorable matters to families	5.39	15	4.77	6.01	***	0.049	5.68	5.12	5.51	**	0.006	5.80	5.33	5.18	***	0.008
Problem behaviors/inadequate behaviors (such as alcoholism and gambling) of your close family members	5.84	11	5.25	6.51	***	0.050	6.06	5.65	5.99	*	0.003	6.13	5.92	5.57	**	0.006
Children are taking Examination and advancing to higher school, or they are preparing to do so	4.71	20	4.29	5.13	***	0.028	4.47	4.52	5.14	***	0.014	5.10	4.57	4.56	***	0.009
Large financial losses or sudden expenditure	6.71	3	6.16	7.32	***	0.041	6.97	6.47	6.89	**	0.005	7.08	6.78	6.35	***	0.010
Family members increase (newborn infant, daughter in law or step parents) or decrease (son or daughter leaves home)	4.63	21	4.30	4.95	***	0.017	4.65	4.74	4.48	ns		4.97	4.55	4.42	***	0.007
Having difficulties to loan repayment	6.33	6	5.83	6.86	***	0.030	6.64	6.20	6.32	ns		6.61	6.51	5.99	***	0.008
Loan or debt about housing and consumption	6.14	9	5.73	6.56	***	0.022	6.60	6.04	5.91	***	0.009	6.39	6.12	6.00	ns	0.002
Major changes in life (such as natural disaster, fire disaster or being involved in crime)	6.52	5	5.89	7.20	***	0.046	6.85	6.27	6.65	**	0.005	6.79	6.75	6.15	***	0.008
Traffic accidents	6.02	10	5.38	6.68	***	0.051	6.18	5.86	6.16	ns		6.56	5.92	5.71	***	0.015
Move house	4.18	23	3.78	4.63	***	0.030	4.26	4.11	4.27	ns		4.34	4.32	3.99	*	0.003
Betrayed by friends	5.27	17	4.82	5.72	***	0.027	5.65	5.14	5.15	**	0.005	5.51	5.20	5.19	ns	0.002
Death of close friend	5.37	16	4.63	6.18	***	0.074	6.03	4.97	5.44	***	0.021	5.72	5.45	5.03	***	0.009
Being disappointed in or bothered (such as extramarital affairs) by love affairs	5.49	14	4.93	6.05	***	0.038	5.92	5.45	5.23	***	0.007	5.81	5.48	5.29	*	0.004
Dispute with neighbors or roommates	4.47	22	3.98	4.99	***	0.038	4.83	4.23	4.56	***	0.008	4.70	4.57	4.24	**	0.005

*p < 0.05; **p < 0.01; ***p < 0.001; ns: not significant

${\text{r}}^{ 2}_{\text{pbi}}$: Square of point-biserial correlation coefficient between “gender” and stress intensity item mean scores; Epsilon^2^: Explanation variation of “age group” and “employment grade” and stress intensity item mean scores according to the calculation formula Epsilon^2^ = dfb(F − l)/dfb + dfw, derived from Hobson et al. [13]

^a^Grade 1: administrators and managers; Grade 2: professionals; Grade 3: non-manual skilled; Grade 4: non-manual low-skilled; Grade 5: manual skilled; Grade 6: manual low-skilled

## Methods

Between 2010–2011, we invited manufacturing and service firms of various sizes located in northern Taiwan to participate in an anonymous questionnaire of employees’ psychological stress levels and physical and mental health. A total of 7 companies agreed to participate; 2 photoelectric and electronics manufacturers, 4 service firms in the hospitality, financial, health care, and social service industries, and 1 government research unit. Regarding sampling methods, all employees in 4 of the firms participated; random sampling of employees was applied for 1 of the firms; and representative employees from numerous departments were purposely selected from the other 2 firms. Researchers collaborated with appointed personnel in the enrolled companies to introduce the study, and to distribute and retrieve questionnaires. In total, 2319 workers participated in the survey (valid return rate 79.3%).

In order to rigorously evaluate the usability of the questionnaire, 6 occupational medicine professionals, psychiatrists and mental health workers were invited to discuss the content validity of the questionnaire prior to commencement of the survey. In addition, 36 workers in the manufacturing, service and government departments were invited to participate in the interview in person to assess the face validity of the questionnaire and to discuss the scoring mechanisms for the stress intensity and the stress situation of the respondents. According to the suggestions from the expert forum, the researchers added 2 work-related stress items, “unpaid leave/leave of absence” and “uncertain career prospects” in response to Taiwan’s recent changes in the workplace caused by the serious global market competition. Researchers also asked the 2 new items in in-depth qualitative interviews and received their approval prior to adding them into the questionnaire survey. Due to the limited length of the paper, detailed content of the preliminary qualitative research results can be found in other research reports [20, 21].

The survey questionnaire was semi-structured and included the following components:

1. Workplace and non-workplace psychological stress: After referring to the SRRS related measurement scales [9, 11, 13–16, 22] and engaging in many discussions with field-related scholars and several pilot-study participants, we modified the “worker psychological stress assessment scale” [19] from the Taiwanese evaluation guidelines into a version more suitable for questionnaire surveying. Certain items with less stress or similar meanings were moderately merged to reduce the scale length. The “workplace and non-workplace psychological stress assessment scales” of this study comprised work-related stress incidents faced by Taiwanese workers (35 items, including “being fired or forced to resign”, “demotion”, etc.), and non-work-related personal life stress incidents (23 items, including “divorce or separation”, “reduced income”, etc.). Tables 1 and 2 show the complete list of items (further details are described elsewhere in Hu and Yeh [20]. Respondents were asked to evaluate the level of stress for each item with scoring ranging from 1 to 10 (lowest to highest stress levels); among them, ≥ 6 points equated to severe stress, 4–6 points to moderate stress, and < 4 points to weak stress. After providing stress scores for every item, respondents were then asked to consider whether those incidents had occurred within the previous year to determine which scores were responses to imagined scenarios. Open-ended questions were also designed to allow respondents to describe other stressful incidents in addition to the specified workplace and non-workplace incidents listed. These were asked for future reference and the results of the resulting qualitative data analysis were published elsewhere [20, 21].
2. Work stress-related symptoms: Burnout status was assessed by the Chinese version of the Copenhagen Burnout Inventory (C-CBI), which included “personal burnout” (5 questions), “work-related burnout” (5 questions), and “client-related burnout” (6 questions) with scores ranging between 0 and 100 points (higher scores indicating greater burnout) [23–25], and the “Chinese Health Questionnaire (CHQ-12),” which included 12 items with scores ranging between 0 and 12 (higher scores indicating more significant mental health issues) [26, 27]. These scales were translated or developed by Taiwanese researchers, and had previously undergone reliability and validation tests, exhibiting good performance.
3. Demographic/socioeconomic background: The questionnaire also included gender, age, level of education, marital status, occupational level, tenure, hours of work per week, and employment status (i.e. permanent employee or temporary worker).

All statistical analyses were performed with SPSS software version 22, and any *p* value < 0.05 was considered statistically significant. The survey depicted the socio-demographic variables of the sample using a descriptive method. These data were used in conjunction with the psychological stress level scale, CHQ-12, and C-CBI to determine sample characteristics. Compared with the “Survey of Perceptions of Safety and Health in the Work Environment in 2007 Taiwan,” completed by the Institute of Occupational Safety and Health, the representativeness of survey respondents was assessed. “Workplace and non-workplace stress level rankings” were also established to determine the relative levels of stress produced by workplace and non-workplace stressors assessed in the survey.

To determine any differences in stress level assessments based on demographic and socioeconomic characteristics, *t*-test and ANOVA analysis were used to compare various stress scores between different groups (gender, age, and employment grade), as well as to assess the proportion of variance in stress scores that can be explained by these variables (explanatory power). This study references the analytical methods employed by Hobson et al. [13] in their research on assessments of stressors. The square of point biserial correlation coefficients, ${\text{r}}^{ 2}_{\text{pbi}}$, was used for dichotomous categorical variables, whereas Epsilon^2^ = dfb (F − l)/dfb + dfw was used for variables with at least three categories.

Finally, to determine the impact of workplace and non-workplace stressors on symptoms of physical and mental illness, this study employed a multiple linear regression analysis and a multiple logistic regression analysis to examine the connection between stress levels after incorporating and controlling for demographic and work characteristic variables frequently used to explain the mental and physical health of workers. This analysis included the following 4 dependent variables related to health consequences: personal burnout, work burnout, and client burnout scores (continuous variables) from the C-CBI, as well as mental health score. The analysis classified respondents scoring ≤ 2 on the CHQ-12 as having “normal mental health” and ≥ 3 as having “poor mental health” based on previous literature on mental health (categorical variable) [28, 29].

The calculation method of the primary independent variable for the multiple regression, “total work-related and non-work-related stress score,” was based on research by Zimmerman et al. [22] on assessments of stressors. The incidents marked by respondents as having been experienced were weighted based on the average stress level scores of the overall sample. Health conditions prediction were then performed based on the resulting sum of stress scores. Thus, this study calculated a composite score of the stress sources experienced within the previous year and compared whether there were differences in the explanatory power of work-related/non-work-related stress levels on personal burnout, work burnout, client burnout, and CHQ-12 mental disorder screening status, after adjusting for potential confounders including demographic and work characteristic variables.

## Results

### Respondent characteristics

Among the 2319 survey respondents, 58.3% were male, 73.8% completed undergraduate or higher education, 46.7% were married, and the mean age was 35.6 ± 9 years. In terms of work characteristics, 56.7% were manufacturing workers, 43.3% were service and public sector workers, 92.6% were full-time employees, the average tenure was 7.8 ± 7.6 years, and average weekly working hours were 53.5 ± 14.5 h. For the items from the “worker workplace and non-workplace psychological stress assessment scale,” (all items are listed in Table 1 and 2) participants reported experiencing 3.1 ± 3.1 work-related incidents and 1.1 ± 1.8 non-work-related incidents on average within 1 year prior to the investigation. The average scores for personal burnout, work burnout, and client burnout were 45.9, 42.0, and 38.1, respectively. The average score for the CHQ-12 in this study was 3.8, which was higher compared to those of Yang’s [28] CHQ-12 mental health study in Taiwanese adults (1.9 on average).

### Rankings of workplace/non-workplace stressors

This study assessed the average stress scores and rankings of 35 work-related and 23 non-work-related stressors examined in this study. The average stress level scores and rankings of stressors for respondents in this study are shown in Tables 1 and 2. The highest-ranked items for work-related stressors were “being fired or forced to resign” and “company bankruptcy.” (6.18 points for both). These items are both related to the continuation of careers. The 2 work-related stress items “unpaid leave/leave of absence” (5.85 points) and “uncertain career prospects” (6.07 points) suggested by the researchers were also high-ranked items for work-related stress. In addition, “a large loss of money at work” (5.69 points) and “being investigated about your responsibility to accidents (events) in company” (5.64 points) were also events associated with high levels of stress in the workplace.

Relatively severe non-work-related stressors included “serious injury, disease or health degeneration to close families” (6.93 points) followed by “death of spouse, child, parent, or sibling” (6.87 points), “large financial losses or sudden expenditure” (6.71 points) and “income decrease” (6.62 points). These are incidents related to changes in the health of close family members or family finances. Other categories, such as relationship or communication problems with family members or family crises were also relatively high-stress non-workplace incidents.

### Demographic and socioeconomic differences in the workplace and non-workplace incidents: gender, age, and occupational level

Analysis of the data indicates that women reported higher stress scores for all workplace and non-workplace incidents than men. Differences in all items were significant, expect for “get married”. The item with the greatest gap in stress levels between the genders was “subjected to sexual harassment”; “gender” explains up to 16.1% of the variance in the stress scores for this item (i.e., ${\text{r}}^{ 2}_{\text{pbi}}$ = 0.161). For age, results showed that workplace incidents such as those related to the continuation of career (including “being fired or forced to resign” and “company bankruptcy”), and “work environment automation and computerization” were more stressful for older individuals than for younger individuals. Conversely, workload or workplace conflicts were more stressful for younger individuals than for older individuals. However, the variance explained by the discussed differences was < 3%. Employing various “employment grade” groups to compare differences in stress levels also showed that incidents related to the continuation of career and workplace conflicts were more stressful for manager-level individuals than for laborers. Non-workplace incidents, such as personal or family health deterioration, financial burdens, and financial losses were also more stressful for manager-level individuals than for laborers. However, the explained variance was also < 3%.

### Multiple regression analysis for the workplace and non-workplace stress with personal burnout, work burnout, client burnout, and physical/mental health of employees

As seen in Table 3, after controlling for demographic and work characteristic variables, individuals who reported high total scores for work-related stress incidents within the previous year had significantly higher personal burnout, work burnout, client burnout, and CHQ-12 scores. There was no significant correlation between the total score of non-work-related stress incidents and scores in personal burnout, work burnout, and client burnout; the only significant influence was on CHQ-12 categorizations. In other words, when controlling other independent variables, an increase in work-related or non-work-related stress scores by 1 point multiplied the odds ratio for CHQ-12 “poor mental health” by e^β^ = 1.03.

**Table 3:** Multivariate regression analysis/logistic regression analysis for work/non-work-related stress intensity scores and demographic/work attributes of personal burnout, work burnout and client burnout measured by Copenhagen Burnout Inventory as well as poor mental health measured by Chinese Health Questionnaire (n = 2319)

	Personal burnout		Work burnout		Client burnout		Poor mental health (CHQ score≧3)		
	β	*p*	β	*p*	β	*p*	β	e^β^(OR)	*p*
Total intensity score of experienced work-related stress items (35 items)^a^	0.20	***	0.22	***	0.23	***	0.03	1.03	***
Total intensity score of experienced non-work-related stress items (23 items)^a^	0.03	ns	0.00	ns	0.02	ns	0.03	1.03	**
Gender									
Male	0		0		0		0		
Female	0.23	***	0.25	***	3.59	*	0.58	1.78	***
Age (years)	− 0.08	*	− 0.12	***	− 0.32	**	− 0.03	0.97	*
Education									
Senior high and below	0		0		0		0		
University/College	0.02	ns	0.06	*	3.08	ns	− 0.22	0.80	ns
Graduate	0.01	ns	0.05	ns	− 0.43	ns	− 0.11	0.90	ns
Marital status									
Married/cohabited	0		0		0		0		
Single/divorced/separated/widowed	0.00	ns	0.01	ns	− 2.27	ns	0.29	1.33	ns
Seniority (years)	0.02	ns	0.00	ns	− 0.05	ns	0.02	1.02	ns
Employment grade									
G1/G2: manager/professional	0		0		0		0		
G3/G4: non-manual skilled/low-skilled	− 0.09	***	− 0.11	***	− 0.21	ns	− 0.54	0.58	**
G5/G6: manual skilled/low-skilled	− 0.05	ns	− 0.40	ns	− 1.25	ns	− 0.29	0.75	ns
Weekly working hour (hours)	0.06	**	0.08	***	0.07	ns	0.01	1.01	ns
Employment status									
Permanent	0		0		0		0		
Non-permanent	− 0.04	ns	− 0.04	ns	− 7.30	**	− 0.25	0.78	ns

*p < 0.05; **p < 0.01; ***p < 0.001; ns: not significant

^a^The incidents marked by respondents as having been experienced during the past year were weighted based on the average stress level scores of the experienced items in the overall sample. This calculation is mainly based on Zimmermann et al. [22]

## Discussion

This study was based on the “Evaluation Guidelines for psychiatric diseases induced by work-related mental stress” announced by the Council of Labor Affairs (the Council was upgraded to the Ministry of Labor Affairs in 2014), Taiwan, in 2009. In this study, items of the work-related and non-work-related incidents in the evaluation guidelines were converted into a mental stress intensity evaluation scale. This is the first report of the application of a work-related psychological disorder evaluation tool in Taiwan. The data from this study are relevant to various industries and provide an epidemiological reference for work-related stress that is of relevance for several purposes including academia, legislation, and the development of workplace procedures and practice.

When establishing Japan’s evaluation guidelines, Japanese researchers referred to the results of workplace surveys to classify and demarcate the stress intensity of work-related/non-work-related SLEs as Levels I, II, or III for group-level reference data. Whether a Level III intensity stress source occurred was the most relevant factor for determining if a person’s psychological disorder could be considered an occupational disease [7–10]. This study refers to the Japanese practice of quantifying and ranking the psychological stress intensity of Taiwanese workers’ common SLEs inside and outside the workplace and comparing the results of event items analysis in the psychological stress assessment scales in Taiwan and Japan. We found that items such as “company bankruptcy,” “being fired or forced to retire,” “suffering from severe unreasonable or ill-treatment, humiliation, or violent behavior,” and “significant financial losses at work,” which received relatively high-stress intensity rankings in this study, were also categorized as Level III intensity in the Japanese evaluation guidelines. This indicates that these are significant workplace mental stress events in both Taiwan and Japan. The item that received the lowest work-related stress ranking in our survey was “work environment automation and computerization,” which was removed from the Japanese evaluation guidelines in the most recent amendment conducted in 2011 [12]. We infer that the rapid development of information technology has been generally considered a necessary trend for management in various industries; for standard enterprises and employees, increasing personal information technology application abilities no longer poses a significant challenge. However, the results of this study showed that for events outside the workplace, greater differences existed between Taiwan and Japan. The events “income decrease,” “having difficulties to loan repayment,” “severe difficulties with immediate family members (including parental/marital relationship),” and “traffic accidents” received an intensity score > 6 and reached level III intensity in our study. However, Japanese evaluation guidelines only listed them as level II intensity. These events may imply different social meanings under the social support system or cultural context of the 2 countries, so the significance of the occurrence of these events differs. Thus, to accurately classify the intensity of SLEs included in the evaluation guidelines, additional practical or empirical evidence must be obtained to help judge the social situations of workers.

By further comparing the results of this study with those of international studies regarding work-related stress epidemiology, we found the “work-related” events that caused higher stress intensities were “being fired or forced to retire,” “company bankruptcy,” and others related to work continuity and job security. This correlated with the results of an outsourced study conducted by the Ministry of Health, Labor, and Welfare in Japan that found that employees were generally highly concerned by “company failure,” ranking this item as the most stress intensive item for work-related events [9, 30]. The participants included in this study were all employed. International work-related stress studies have found that although a worker may be employed, if they “perceive” that their job security is threatened, adverse mental and physical health developments occur (including depression, anxiety, insomnia, and cardiovascular diseases) [31–33]; this type of anticipatory mindset often produces a greater amount of pressure or stress than that experienced by people who are unemployed. Precarious employment has been indicated as an emerging social determinant affecting workers’ health, and research indicates that further development of the pathogenesis mechanisms will become increasingly important in the next decade [34, 35].

Regarding non-work-related events, analysis of the research results shows that the events “serious injury, disease or health degeneration to close families,” “death of a spouse, children, parents or sibling,” “income decrease,” “large financial losses or sudden expenditure,” and other related events concerning the loss of life/health of a loved one or financial difficulties within the family, caused the highest stress intensities. The intense psychological stress caused by a loss and the adjustment after the loss of life/health of a loved one echoed with the results of earlier studies conducted in Japan [30] and the United States [11, 13, 18]. However, after comparing our results with those of international studies, we found that the participants of this study also experienced a significant amount of mental stress regarding economic difficulties, demonstrating the effect of this stress on workers. Allowing for methodological differences in international studies, there is a possibility that this problem may be particularly significant in Taiwan. This may indicate that for the population in Taiwan, the economy is a particularly significant or sensitive mental stressor. Thus, recognition and support, especially of public policies and organizational management, is essential.

To understand whether significant differences existed in the stress evaluation of various events by various demographic/work characteristics, the stress intensity scores were categorized according to “gender,” “age,” and “employment grade” (G1/G2: administrators, managers, and professionals; G3/G4: non-manual skilled/low-skilled; G5/G6: manual skilled/low-skilled) for group comparisons. The results indicated that among such 3 categories, “gender” showed the most significant difference. For all event items in and outside of the workplace that showed a significant difference according to gender, females experienced greater stress than males. This phenomenon is relatively consistent with the trends observed in Japanese and Unites States investigations [13, 30], and is also similar to the previous studies of gender differences in personal and work-related burnout [24, 25, 36]. The event item that showed the greatest inter-gender difference was “sexual harassment.” This result also correlated with the results for the event in Unites States studies [13]. Therefore, we deduced that sexual harassment in the workplace typically involves the social context or scenarios of gender discrimination and unequal authority, which are products of or found within a patriarchal culture and ideology. This means that for sexual harassment, women showed a higher probability of experiencing this event as well as a greater degree of injury compared to men; thus, this event poses a higher psychological threat. Considering the participants of this study, fewer women held management positions compared to men (6.7% vs. 12.2%, data not shown). This partially reflected the lower authority women have compared to men in the workplace and society. For stress sources in and outside the workplace, such as sexual harassment, women may comparably lack the authority and resources to respond appropriately. The differences for in and outside workplace stress experienced by various age groups had no observable or consistent trend. This result also correlates with that of similar studies conducted previously [13, 30]. It is likely that workplace stress concerns are different for workers of different ages; we infer that for younger individuals, stress was more pronounced when adapting to the workplace or work demands that required experience, but for older individuals, job security and retirement-related benefits were more significant issues. Regarding occupation level, in contrast to previous studies [13, 30] that showed greater stress problems experienced by blue-collar workers versus supervisors and white-collar workers, the results of this study indicated that workers at management level experienced higher mental stress. This trend correlated with the analysis results of previous national surveys in Taiwan [36, 37]. We suppose that high-level workers often experience greater responsibilities at work; thus, they experience greater stress from events for which they are possibly responsible, and their concerns regarding their physical health and economy and finances may be greater. Additionally, it must be noted that although the results showed differences in stress intensity for the previously mentioned demographic groups, these differences should be further quantified with an “explanatory effect amount or power.” Besides “gender” having an explanatory power of 16% for “sexual harassment in the workplace,” the stress intensities of other events in or outside the workplace showed explanatory powers of < 10% for the various demographic categories. We recommend that for future applications of the recognized or recognition guidelines, in addition to considering the group average stress intensity of sexual harassment events in the workplace, a separate and weighted evaluation of the stress experienced and perceived by individual women should be conducted. For other events, specialized processing of stress intensity evaluations according to various demographic categories are less necessary.

Stress is caused by numerous factors in and outside the workplace; the analytical results provided in this study were mainly based on single events. This may limit the applicability of these results for evaluating the overall stress intensity experienced by a person in and outside the workplace. The evaluation guidelines from Japan originally only evaluated whether a person had experienced work-related events with a Level III mental stress intensity when assessing work-related stress; however, the accumulative nature of stress must also be considered. At the last amendment in 2011 [12], for individuals who experienced multiple Level II events for workplace stress, if major stress outside the workplace and personal factors were excluded, they could be considered to have experienced occupational injury due to overwork or overloading. For this study, we also referenced the stress intensity calculation method developed by Zimmermann et al. [22] to determine a stress score for SLEs experienced in or outside the workplace. We found that the influence of stress on psychosomatic symptoms due to events in and outside the workplace was similar, but SLEs experienced in the workplace showed a stronger relationship with subjective burnout conditions compared to SLEs experienced outside the workplace. From the results of this study, the cumulative effect of multiple stressors on the causes of mental illness cannot be ignored in the identification of whether mental illness is work-related.

Although this series of studies considers breadth (questionnaire survey) and depth (qualitative interviews, expert meetings) to investigate the applicability of Taiwan’s identification guidelines, there are still some limitations in the current study. First, due to the cross-sectional design, we were unable to determine the causal relationship between stress and symptoms (despite in the invitation process of study participants, those diagnosed with a mental illness were excluded). Furthermore, in this study, the contents of the questionnaire was subject to the identification guidelines. We propose to consider further the theory of work stress and life events measurement to continuously improve the content and scoring method for major SLEs as well as daily hassles in the follow-up study. In addition, it would also be worthwhile mentioning the fact that different sampling methods were used from different participating firms in the study, which raises the potential for selection bias. Comparing the characteristics of our study participants with the wider Taiwanese worker population [37] for age, gender, and education level showed that the participants of this study were comparatively younger, more educated, worked longer, with 90% of them officially or permanently employed. Compared to the results published by Yang [28] regarding the CHQ-12 psychiatric conditions of adults in Taiwan, the psychiatric condition of the participants in this study showed significantly higher scores. These participants may represent a subgroup of nationwide employees who have experienced greater work-related stress. The theoretical inferences of the results of this study for people with lower education levels, temporary or non-official employment, or who are self-employed (such as employers or people who run their own businesses) are limited. The participant diversity of this preliminary study was achieved by conducting supplementary qualitative in-depth interviews with workers from a wider scope [21]. We recommend that a subsequent study expand the research to include workers from a greater number of industries and employment identities to increase the research sample’s representativeness of the national employee population.

## Conclusions

Despite the limitations described, this study provides population-level quantitative survey data for the system of accrediting psychological disorders as an occupational injury. By conducting interviews with workers and utilizing related content, people’s mental stress intensity levels were determined and the significant contributing factors analyzed. The results can be used by those employed in the occupational medicine field and psychiatrists to facilitate clinical diagnosis of whether psychological disorders are caused by work-related stress. The results of this study indicate that when applied to Taiwan, the stress intensity levels of certain events differed from the values listed in the Japanese evaluation guidelines. Furthermore, we also identified several other important SLEs that occur in and outside the workplace in Taiwan. We anticipate that the results of this study will be used as a reference point for future developments and amendments of the recognized or recognition guidelines. Besides their significance for occupational health policies, the results of this study are also valuable for industries. By understanding the common or concern-inducing SLEs experienced by employees, companies can appropriately develop and prioritize employee support protocols and services. Thus, all available resources can be utilized with the greatest efficiency, and the health risks caused by employees’ mental stress can be effectively managed.

## Data Availability

The data sets supporting the results of this article are included within the article and its additional files.

## Abbreviations

SRRS: Social Readjustment Rating Scale
SLEs: stressful life events
CBI: Copenhagen Burnout Inventory
CHQ-12: Chinese Health Questionnaire (12-item version)
